# Marine-Derived Metabolites of *S-*Adenosylmethionine as Templates for New Anti-Infectives

**DOI:** 10.3390/md7030401

**Published:** 2009-08-26

**Authors:** Janice R. Sufrin, Steven Finckbeiner, Colin M. Oliver

**Affiliations:** Department of Pharmacology and Therapeutics, Grace Cancer Drug Center, Roswell Park Cancer Institute, Buffalo, New York, NY, USA; E-Mails:steven.finckbeiner@roswellpark.org (S.F.);colin.oliver@roswellpark.org (C.O.)

**Keywords:** adenosylmethionine, ethylene, polyamines, radical SAM, quorum sensing

## Abstract

*S*-Adenosylmethionine (AdoMet) is a key biochemical co-factor whose proximate metabolites include methylated macromolecules (*e.g.*, nucleic acids, proteins, phospholipids), methylated small molecules (*e.g.*, sterols, biogenic amines), polyamines (*e.g.*, spermidine, spermine), ethylene, and *N*-acyl-homoserine lactones. Marine organisms produce numerous AdoMet metabolites whose novel structures can be regarded as lead compounds for anti-infective drug design.

## 1. Introduction

*S*-Adenosyl-l-methionine (AdoMet, SAM) is a biochemical intermediate which serves as precursor to a vast compendium of bioactive metabolites in all living organisms. The remarkable structural diversity that characterizes known metabolites of AdoMet is a key component of AdoMet’s ability to meet specialized needs in highly different microenvironments. Many AdoMet metabolites and AdoMet-utilizing pathways, as yet unknown, remain to be discovered and characterized. Marine environments provide an attractive and abundant reservoir to search for new classes of molecules that are structurally derived from AdoMet. This is corroborated by a recent novel finding that the chloro-substituent of salinosporamide A, a marine product now undergoing Phase I clinical trials for cancer treatment, is enzymatically derived from AdoMet [1].

The aims of this article are: 1) to present an overview of AdoMet metabolism that increases readers’ awareness of the intrinsic roles of these pathways in maintaining the viability of all living organisms; and 2) to highlight novel marine natural products that incorporate structural components of AdoMet in their molecular structure. Our intent is to provide broadly based evidence for our premise that marine environments will remain a continuous source of new AdoMet metabolites that can be viewed as templates for novel anti-infectives and other therapeutic agents.

## 2. Overview of Major AdoMet-Utilizing Pathways

**Figure 1:**
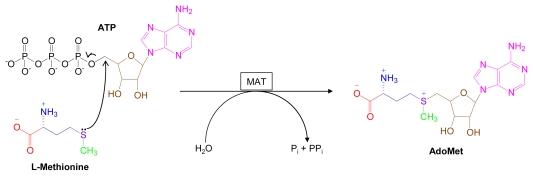
*S*-Adenosylmethionine biosynthesis. Structural components of AdoMet are color coded.

AdoMet is synthesized from l-methionine and ATP by various isoforms of the enzyme, methionine adenosyltransferase (MAT, Figure 1) [2]. Central to AdoMet’s reactivity is the presence of a chiral sulfonium ion whose three adjacent alkyl substituents are susceptible to nucleophilic attack at their respective carbon-sulfur bonds [3,4]. A robust family of methyltransferases catalyzes transfer of AdoMet’s methyl group to diverse biological substrates [5]. Nucleophilic attacks directed at the carbon-sulfur bond of ribose lead to formation of 5’-halogenated adenosine derivatives [6]; those directed at the 3-amino-3-carboxypropyl portion of AdoMet give rise to polyamines, ethylene and homoserine lactone derivatives [3,4].

As the principal biological methyl donor, AdoMet is an obligatory cofactor for the enzymatic methylation of DNA, RNA, proteins, phospholipids, and various small molecules such as catecholamines, steroids, etc. Polyamine biosynthesis is another important AdoMet-dependent pathway: AdoMet, subsequent to its enzymatic decarboxylation, serves as aminopropyl donor for synthesis of the ubiquitous polyamines, spermidine and spermine. Ethylene, another key metabolite of AdoMet, is produced in plants where it plays major roles in ripening, senescence and responses to stress. AdoMet-dependent biosyntheses of methylated molecules, polyamines and ethylene have been studied for many years and are regarded as classical pathways of AdoMet metabolism.

In more recent years, two major pathways of AdoMet metabolism in bacteria have been uncovered. The discovery of *N*-acyl-l-homoserine lactones, a new class of signal molecules produced by many gram negative bacteria, was followed by studies which determined that its l-homoserine lactone component is enzymatically derived from AdoMet [7,8]. Autoinducer-2 is another important bacterial signal molecule which is derived from AdoMet [9].

A new class of AdoMet-utilizing proteins, the radical SAM superfamily, was discovered recently [10]. These proteins use AdoMet as substrate or catalyst in various enzymatic reactions that are associated with a diverse array of chemical transformations and biological functions [10–12]. In addition, a novel role for AdoMet in the biosynthesis of complex, halogenated organic molecules was first reported in 2002 [13,14]. Subsequently, an AdoMet-utilizing halogenase was identified in a marine organism [1]. The importance and scope of AdoMet-dependent biohalogenation pathways will become more evident as additional scientific studies are published.

Several uncommon pathways of AdoMet metabolism are worthy of mention because they highlight novel, unprecedented donor properties of this ubiquitous sulfonium compound [4]. AdoMet serves as amino donor for biosynthesis of 7,8-pelargonic acid, an intermediate in the biosynthesis of biotin [15]. AdoMet serves as 3-amino-3-carboxypropyl donor for biosynthesis of the hypermodified nucleoside, 3-(3-amino-3-carboxypropyl)uridine, which was first found in *Escherichia coli* tRNA [16–18]. AdoMet serves as ribosyl donor for biosynthesis of the hypermodified tRNA nucleoside, queuosine [19]. These highly specific, AdoMet-dependent, structural modifications to RNA are complemented by AdoMet’s more pervasive activities as methyl donor for RNA [20–25].

## 3. AdoMet Pathways and Marine-Derived AdoMet Metabolites

The role of AdoMet as precursor for ethylene, polyamines and methylated molecules has been extensively documented in the scientific literature. Biochemical studies with labeled precursors have been instrumental in validating the diverse pathways of AdoMet consumption in living organisms. For the most part, these studies have used terrestrial organisms as platforms for experimental design. Discovery of the AdoMet-derived, bacterial signal molecules, *N*-acyl-l-homoserine lactones (AHLs) and autoinducer-2 (**AI-2**), in marine bacteria is a notable exception.

**Figure 2:**
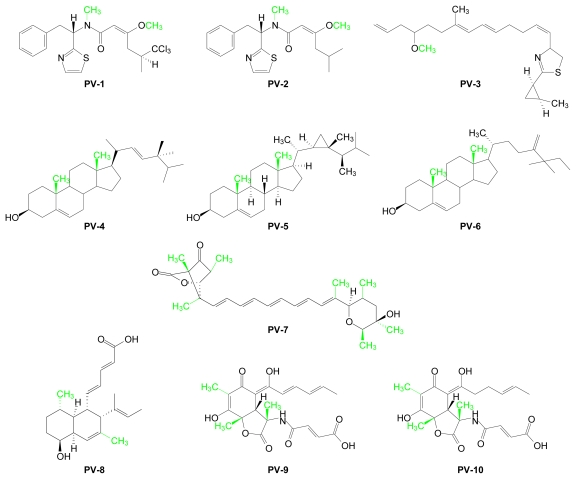
Structures of marine-derived, precursor-validated AdoMet metabolites. AdoMet-derived methyl groups are shown in green.

Precursor studies using marine organisms have been reported but their scope has been limited by the elusiveness of marine microorganisms and challenges associated with scientific exploration of marine habitats. When such studies were carried out, most were designed to provide evidence of the role of AdoMet as the biological methyl donor for a range of marine-derived methylated natural products. Several examples of marine-derived molecules whose methyl group origins have been unambiguously attributed to AdoMet by labeled methionine precursor studies are listed in Table 1. Their structures are shown in Figure 2.

Labeled decarboxylated AdoMet was used to determine that AdoMet is the source of aminopropyl groups of the polyamines produced by *Pyrococcus furiosus*, a hyperthermophilic archeon [36]. Moreover, these studies identified a new aminopropyl transferase enzyme whose natural substrates include the expected 1,4-diaminobutane (putrescine) as well as the less common diamines, 1,3-diaminopropane, cadaverine, thermine and agmatine [36].

### 3.1. Polyamine Pathways [37–44]

**Figure 3:**
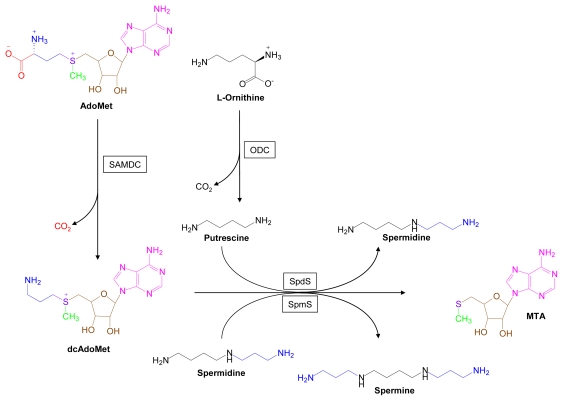
Polyamine Biosynthesis. Structural components derived from AdoMet are color coded. SAMDC, *S*-adenosylmethionine decarboxylase; ODC, ornithine decarboxylase; SpdS, spermidine synthase; SpmS, spermine synthase.

Putrescine, spermidine and spermine are the major eukaryotic polyamines. Putrescine, which is enzymatically derived from ornithine, is metabolized to spermidine and then to spermine by successive enzymatic transfers of an aminopropyl group from decarboxylated AdoMet (Figure 3).

The purine nucleoside, 5’-deoxy-5’-(methylthio)adenosine (MTA) is the common byproduct of spermidine and spermine synthesis. At physiologic pH, polyamines exist as polycations and modulate functions of acidic structures such as DNA, RNA, phospholipids and proteins. They are important participants in processes associated with cell viability, growth and differentiation. The naturally high intracellular levels of polyamines (*i.e.*, mM concentrations) have made clarification of their high affinity molecular targets more difficult. They are known to specifically affect the functions of ion channels and the *N*-methyl-d-aspartate (NMDA) glutamate receptor at physiological concentrations [40,45]. Igarashi and colleagues studied the binding interactions of spermidine and spermine with cellular DNA, RNA, phospholipids and ATP in rat liver and bovine lymphocytes [41]. In both cell types, the largest fractions of intracellular spermidine and spermine were found to be associated with RNA, suggesting that the structural changes to RNA arising from these binding interactions may play a major role in the intracellular functions of polyamines [41].

Polyamines are involved in key cellular functions such as responses to oxidative stress, pH, and osmoregulation, which are of particular importance to marine bacteria. These functions are even more important to extreme thermophilic bacteria, which use polyamines to stabilize their RNA and DNA at high temperatures [46]. The fact that polyamines have specialized functions in aquatic environments is evidenced by an abundance of novel straight chain and branched polyamines produced by marine organisms. The marine thermophile, *Thermus thermophilus* is an example of a prolific producer of different polycationic polyamine structures (shown in Table 2).

A collection of unusually long-chain polyamines (LCPAs) has been isolated from the marine sponge, *Axinyssa aculeate*. A composite of their structures, ^+^H_3_N-(CH_2_)_3_-[NH_2_^+^-(CH_2_)_3_]n–NH_3_^+^ (n = 4–14), gives an idea of their extraordinary length [47]. Other LCPAs have been isolated from marine algae [48–50]. LCPAs are known to combine with silica precipitating proteins (silaffins) to produce a composite material called biosilica that is essential to the formation of complex cell wall structures such as those found in shells. Biomineralization is manifested by the use of species-specific sets of silaffins and LCPA constituents whose structural variations may include differences in chain length, *N*-methylation patterns and/or the positioning of secondary amino substituents and quaternary ammonium groups [48,49,51]. Consequently, polyamines are essential for the formation of intricate silica patterns on cell walls of diatoms [47–50]. From a technical perspective, an understanding of the biochemical mechanisms that regulate nanoscale production of biosilica-based structures is highly relevant to research and product development in the field of nanotechnology [51].

**Figure 4:**
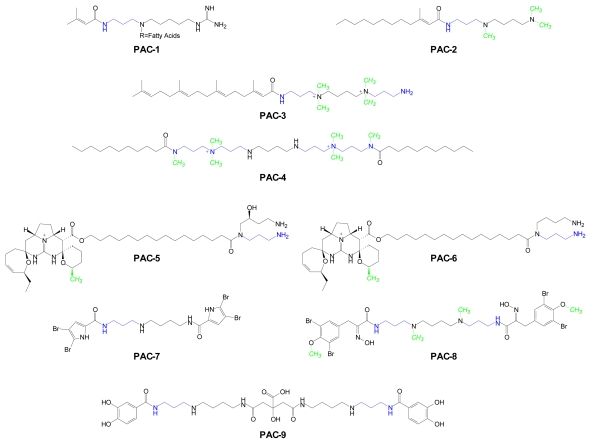
Structures of marine-derived polyamine conjugates. AdoMet-derived aminopropyl (blue) and methyl (green) groups are depicted.

Marine habitats have also proved to be a rich source of unusual polyamine conjugates (PACs) such as those depicted in Figure 4. For each conjugate, at least one marine source is listed in Table 3. Crambescidin 800 (**PAC-5**) and ptilomycalin A (**PAC-6**) belong to a family of guanidine alkaloids whose structures contain an unusual pentacyclic guanidine framework linked by a ω–hydroxy fatty acid to a spermidine or hydroxyspermidine moiety. The structures of **PAC-5** and **PAC-6** differ only by the presence or absence of a hydroxyl substituent on spermidine. Although both compounds showed activity in various antitumor and antimicrobial screens, their potencies were unremarkable [53–56]. However, in the course of comprehensive, high-throughput screens of ~3,100 compounds from NCI libraries and >300 crude marine-derived extracts for antifungal activity, Crambescidin 800 emerged as the most potent compound [57].

Penaramide A (**PAC-4**) is one of several acylated polyamine structures that were isolated from the sponge *Penares* aff. *Incrustans*. Penaramides are symmetric molecules that differ only in the composition of their *N*-terminal acyl substituents. Penaramide A, the simplest of these compounds, contains two linear, C11 fatty acids. At the time these compounds were described, they were found to inhibit binding of the peptide neurotoxin, ω-conotoxin GVIA to N-type (high voltage-activated) calcium channels [33].

Acarnidines (**PAC-1**), another class of polyamine fatty acid conjugates, are distinguished by the presence of a homospermidine backbone. As seen in the penaramide series, acarnidines differ only by the structures of their respective fatty acid components. The acarnadines were reported to have significant antimicrobial activity against *Herpes simplex* type 1, *Bacillus subtilis,* and *Penicillium atrovenetum* [58]. Another fatty acid polyamine conjugate, sinulamide (**PAC-3**), is an inhibitor of H,K-ATPase [66]. Sinulamide has structural features similar to some of those seen in penaramides and acarnidines.

The novel alkaloid, spermatinamine (**PAC-8**) is a symmetrical spermine conjugate whose uncommon feature is its unusual acyl component which is derived from 3,5-dibromotyrosine. Spermatinamine is an inhibitor of isoprenylcysteine carboxyl methyltransferase (ICMT), one of the enzymes involved in activation of the Ras signaling pathway [64]. Ras family proteins contain a CAAX terminal sequence that undergoes a series of successive posttranslational modifications, resulting in the translocation of these proteins to the cell membrane [67]. The specific enzymes that contribute to activation of Ras signaling are considered to be promising anticancer targets. Spermatinamine, the first natural product known to inhibit ICMT, is a compound of significant chemotherapeutic interest [64].

Petrobactin (**PAC-9**) was first isolated from *Marinobacter hydrocarbonoclasticus* [68]. This oil-degrading molecule has since been found in both pathogenic and nonpathogenic bacteria [69]. Petrobactin is required for expression of virulence by *Bacillus anthracis*, the causative agent of anthrax disease and is the primary siderophore produced by this pathogen under conditions of iron starvation [65]. Elucidation of its structure, biosynthetic origins and biological properties as well as chemical routes to its synthesis, have been well established [65,70–81]. Pseudoceratidine (**PAC-7**), an antifouling agent that can prevent attachment of marine organisms (*e.g.*, mollusks, barnacles) to hulls of ships and other submerged structures, was first isolated and synthesized in 1996 [63,82]. Its interesting spectrum of antimicrobial and marine biocidal effects are of potential industrial significance [83]. Trimethylspermidine amide (**PAC-2**) and sinulamide (**PAC-3**), a potent inhibitor of H,K-ATPase, were isolated from different species of the soft coral *Sinularia* [66]. Marine **PACs** with unusually complex *N*-acyl components can be viewed as lead structures for combinatorial synthesis of novel **PACs** from libraries of acyl substituents and linear polyamines.

### 3.2. Methylation Pathways

**Figure 5:**
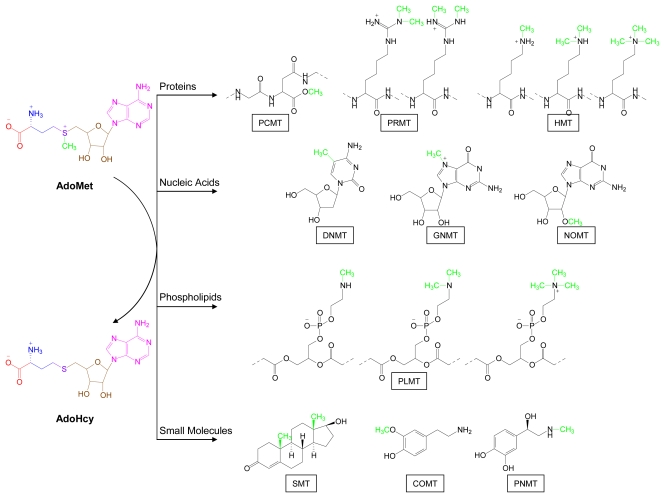
Biological methylation pathways. AdoMet-derived methyl groups are shown in green. AdoHcy, *S*-adenosylhomocysteine; PCMT, protein carboxymethyltransferase; PRMT, protein arginine methyltransferase; HMT, histone methyltransferase; DNMT, DNA methyltransferase; GNMT, guanosine *N*-methyltransferase; NOMT, nucleoside *O*-methyltransferase; PLMT, phospholipid methyltransferase; SMT, sterol methyltransferase; COMT, catechol *O*-methyltransferase; PNMT, phenylethanolamine *N*-methyltransferase.

Methylated molecules are the most abundant type of AdoMet metabolites in living organisms. Their biosynthesis is catalyzed by a superfamily of methyltransferase enzymes [5,84–87] (Figure 5). Several classes of methyltransferases have been structurally defined [5, 86–88]. The AdoMet binding regions in many of these enzymes contain a common, three-dimensional structural motif that has been used to seek out putative methyltransferases among proteins of unknown function [84]. Clarke and colleagues established a methyltransferase-specific database and have continued to search for protein sequences predictive of methyltransferase function by scanning open reading frames of genomes using automated methods they developed for this purpose [84].

**Figure 6:**
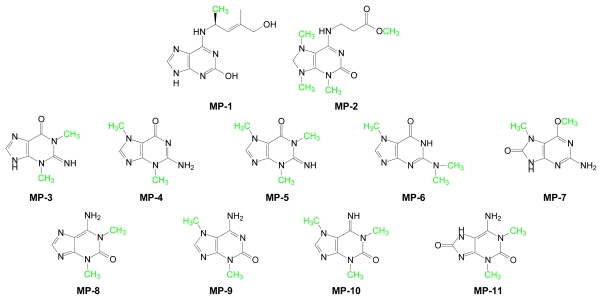

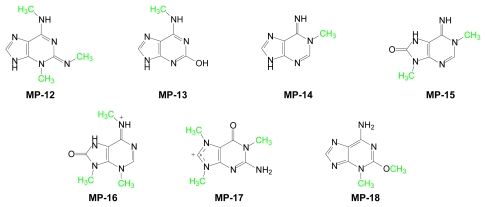
Structures of marine-derived methylated purines. AdoMet-derived methyl groups are shown in green.

**Figure 7:**
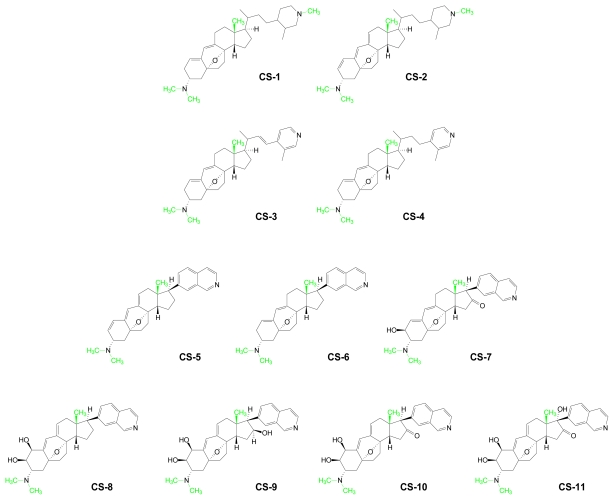
Cortistatins (CS). AdoMet-derived methyl groups are shown in green.

The physiological consequences of enzymatic methyl group transfer can be viewed from fundamental chemical and biochemical perspectives. A methyl group can be transferred to atoms such as carbon, nitrogen, oxygen, sulfur, and selenium. However, methyltransferases that catalyze methyl transfer to carbon, nitrogen and oxygen atoms are predominant. Methyl group addition increases steric bulk and can alter charge, conformation and/or tertiary structure of the acceptor molecule. Furthermore, methylation can profoundly affect biochemical pathways and physiological processes by altering the binding affinities of associated ligands for macromolecules such as proteins, DNA, RNA and phospholipids as well as for small molecule ligands, such as steroids, amino acids, nucleosides and biogenic amines. Complex methylation patterns are seen in many classes of small marine-derived molecules such as purines (Table 4, Figure 6) and sterols (Figure 7).

A variety of methylated purines has been isolated from marine organisms [106]. Figure 6 depicts a selected number of these structures.

Examples of methylation within the purine scaffold, which contains four heterocyclic nitrogen atoms, as well as on some of the exocyclic amino- and imino-substitutents are depicted. **MP-7** and **MP-17** contain exocyclic methoxy substituents. **MP-8** elicits an antitumor response, **MP-9** displays antibacterial behavior and **MP-17** is a collagenase inhibitor [106]. Whether these purine analogs afford any benefits to their host organisms is unclear. However, they may be useful as anti-metabolite templates for potential anti-infectives.

Sponges are the most abundant marine source of novel sterols [107]. Changes in the compositions of these membrane constituents, which are vital for cell permeability, are associated with increased defensive capabilities [108,109]. Marine sterols exhibit structural complexities that are not observed in terrestrial organisms [110]. Although most variations occur in the side chain, the steroid ring system is also subject to chemical transformations [111]. Structural variations also arise in the methylation patterns of steroid rings, alkyl side chains and/or exocyclic substituents. The structurally complex, anti-angiogenic cortistatins isolated from the sponge *Corticium simplex* contain both *C*- and *N*-methylated substituents [112] (Figure 7).

### 3.3. AdoMet-Dependent Ethylene Biosynthesis [113–117]

**Figure 8:**
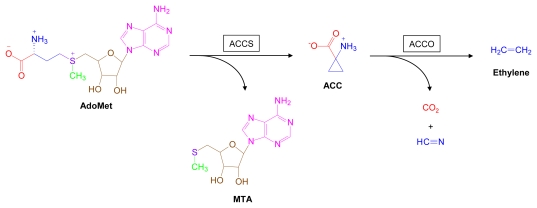
Ethylene biosynthesis. Structural components derived from AdoMet are color coded. ACC, 1-aminocyclopropane-1-carboxylate; ACCS, 1-aminocyclopropane-1-carboxylate synthase; ACCO, 1-aminocyclopropane-1-carboxylate oxidase.

Ethylene is a phytohormone that stands at the apex of a robust signaling pathway in plants. Ethylene, which is enzymatically derived from AdoMet (Figure 8), serves as a critical regulator of life sustaining processes such as plant growth and development, responses to external stresses, and senescence. The terrestrial plant, *Arabidopsis thaliana* has served as a model system for elucidating the biochemical, molecular and genetic complexities of the ethylene signaling pathway, which is still the focus of intense scientific study [84,115,116,118].

AdoMet-dependent ethylene biosynthesis has been documented in a variety of marine plants and sponges [119–121]. *Suberites domuncula*, a marine sponge, responds to the presence of ethylene (5 μM) by upregulating its intracellular concentration of Ca^+2^ and reducing its apoptotic response to starvation [122]. When the marine macroalga *Ulva (Enteromorpha) intestinalis* moves from conditions of low light intensity to high light intensity, its production of ethylene increases. This suggests that ethylene is involved in an adaptive response to light stress [119]. Ethylene is also naturally present in seawater as a consequence of widespread photochemical degradation of organic materials. Thus ethylene can be acquired from the aquatic environment by ethylene-responsive marine organisms that might not contain the biosynthetic machinery for its production.

### 3.4. Biohalogenation Pathways [123–125]

The discovery of a fluorinase enzyme that catalyzes the formation of a carbon-fluorine bond not only opened a new chapter in the field of biohalogenation, but also uncovered a previously unknown pathway of AdoMet metabolism [13]. The fluorinase was first isolated from the soil bacterium *Streptomyces cattleya*. The enzyme’s x-ray crystal structure, catalytic mechanism and kinetic features have since been determined [13,123,126]. The fluorinase reaction yields the proximate AdoMet metabolite, 5’-deoxy-5’-fluoroadenosine, which is ultimately transformed to a toxin, monofluoroacetic acid and an unusual amino acid, 4-fluorothreonine.

**Figure 9:**
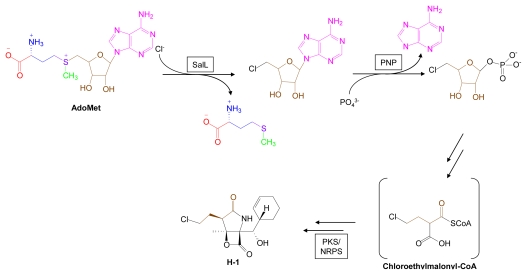
AdoMet-dependent halogenation pathways. A halogen is enzymatically transferred to AdoMet, releasing methionine to generate 5’-halo-5’-deoxyadenosine. SalL, AdoMet-dependent chlorinase; PNP, purine nucleoside phosphorylase; PKS/NRPS, polyketide synthase/nonribosomal peptide synthetase.

Subsequent discovery of an AdoMet-utilizing chlorinase from *Salinispora tropica* has demonstrated the existence of AdoMet biohalogenation pathways in marine organisms [1]. The chlorinase reaction, mechanistically similar to that of the *S. cattleya* fluorinase, produces the proximate AdoMet metabolite, 5’-deoxy-5’-chloroadenosine which is a key intermediate in the biosynthesis of salinosporamide A (Figure 9). Cell-free assays of *S. tropica* chlorinase activity determined that inorganic bromide and iodide, but not fluoride, can be used as inorganic substrates in place of chloride, suggesting that brominated and iodinated marine structures arising from AdoMet-dependent biohalogenations may possibly be found in the future [1].

### 3.5. Radical SAM Pathways [10–12,127–130]

In a groundbreaking study, Sofia and colleagues discovered a new protein superfamily of enzymes, designated radical SAM, through iterative profile searches of protein databases, data analysis employing powerful bioinformatic tools and information visualization techniques [10]. Shared features of radical SAM proteins are the presence of an uncommon iron-sulfur cluster [4Fe-4S], the specific sequence motif CxxCxxC and an AdoMet binding motif.

**Figure 10:**
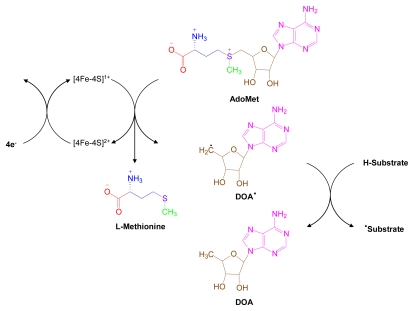
. Radical SAM pathways [11,127,128]. AdoMet is utilized as a protein cofactor or catalyst in radical SAM reactions. These proteins contain an embedded iron-sulfur cluster that binds AdoMet, releases methionine and generates a deoxyadenosine (DOA) radical. The DOA radical transfers an electron to the enzyme substrate to generate a substrate radical. Structural components derived from AdoMet are color coded.

Two basic types of radical SAM proteins have been characterized. One uses AdoMet as a catalytic cofactor that is the direct precursor of the 5’-deoxyadenosyl radical (DOA radical). The other uses AdoMet as a substrate to irreversbly generate a DOA radical. In the first reaction type, AdoMet abstracts a hydrogen atom from 5’-deoxyadenosine (DOA), the end product of step 1, and concomitantly, recycles AdoMet and regenerates a DOA radical. In the second type, AdoMet serves as a radical SAM substrate whose proximate products are methionine and a DOA radical. The initial reaction steps, which are identical for all SAM enzymes, are illustrated in Figure 10.

DOA radicals can act as powerful anaerobic oxidants whose biochemical functions vary among different radical SAM proteins. Many of these proteins use DOA radicals to cleave and functionalize otherwise unreactive carbon-hydrogen (C-H) bonds in protein and small molecule substrates [12]. Chemical reactions such as isomerization, sulfur insertion, dehydrogenation and cyclization are catalyzed by radical SAM family members [10,11]. The radical SAM enzymes, biotin synthase and lipoyl synthase are responsible for the production of the key biochemical cofactors, biotin and lipoic acid [11,12,127–130]. Brief mention here of other known radical SAM family members attests to the functional diversity of these proteins: spore photoproduct lyase; anaerobic ribonucleotide reductase activating enzyme; benzylsuccinate synthase; coproporphyrinogen III oxidase; lysine 2,3-amino-mutase; pyruvate formate-lyase [127].

Existence of radical SAM proteins in marine organisms has been noted. The green sulfur bacterium**,** *Chlorobaculum tepidum*, produces bacteriochlorophylls *c, d*, and *e*, a group of photosynthetic pigments that differ from other chlorophylls by the presence of methyl groups at their *C*-8^2^ and *C*-12^1^ carbons. These site-specific methylations are critical to the organism’s ability to adapt to decreased light intensities [131]. Labeled precursor studies confirmed that these methyl groups are derived from AdoMet [132]. The methyltransferases responsible for addition of these two methyl groups have been identified recently as members of the radical SAM superfamily [131]. Sequencing of the complete genome of *Acaryochloris marina* has led to the discovery of twelve proteins that contain the characteristic, distinguishing features of radical SAM proteins [133]. *A. marina* is a cyanobacterium whose predominant photosynthetic pigment is chlorophyll *d*. The presence of this unusual pigment enables *A. marina* to use far-red light for its photosynthetic pathways.

### 3.6. Quorum Sensing Pathways

**Figure 11:**
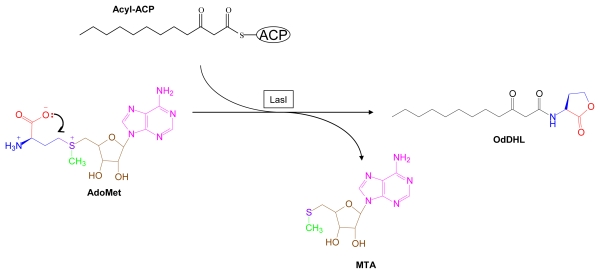
*N*-Acylhomoserine lactone biosynthesis. Structural components derived from AdoMet are color coded. LasI, AHL synthase. OdDHL, *N*-3-oxo-dodecanoyl-l-homoserine lactone.

Some gram-negative bacteria sense conspecific cell density, and the density of other bacteria, by monitoring the extracellular concentration of specific small molecules. This phenomenon, called quorum sensing (QS), is used by bacteria to coordinate transcriptional regulation of genes that control population-sensitive programs. QS was first described as a general phenomenon in a landmark review [134]. An example from this review illustrates the process as a whole: *Vibrio fischeri*, a bioluminescent marine bacterium, expresses genes needed to produce bioluminescence only at high population density. A small molecule, *N*-3-oxo-hexanoyl-l-homoserine lactone, accumulates in culture fluid with increasing density of *V. fischeri. N*-3-oxo-hexanoyl-l-homoserine binds to the receptor/transcription factor, LuxR, which controls population density dependent expression of bioluminescence genes. Further work showed that different species of bacteria use different AHLs of varying acyl chain lengths as species specific QS signals. In addition to control of bioluminescence in other *Vibrio* species, AHL-controlled QS coordinates what are effectively multicellular developmental programs in a wide range of bacteria. These include biofilm formation, swarming, and induction of virulence in pathogenic species. [135–137]. AHLs are enzymatically produced from AdoMet and an acyl-acyl carrier protein [7,138] (Figure 11).

**Figure 12:**
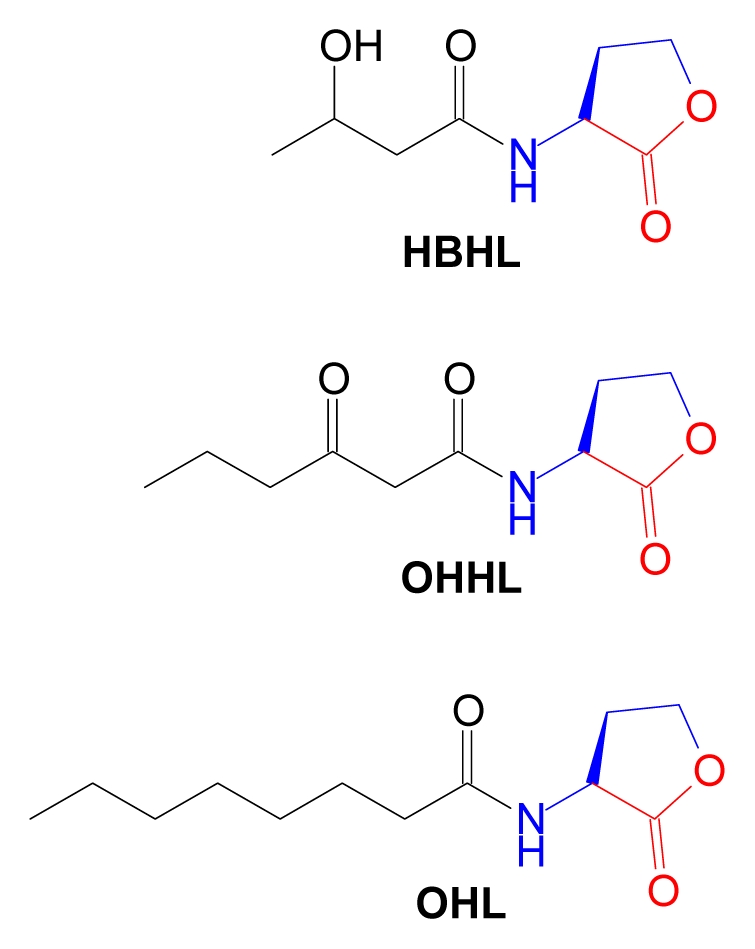
Acylhomoserine lactones from marine bacteria genus *Vibrio*. Structural components derived from AdoMet are color coded. HBHL, *N*-3-hydroxy-butanoyl-l-homoserine lactone; OHHL, *N*-3-oxo-hexanoyl-l-homoserine lactone; OHL, *N*-octanoyl-l-homoserine lactone.

The lactone ring is an invariant feature of AHLs, arising from cyclization of the methionine moiety of AdoMet by AHL synthase enzymes. Natural AHL structures vary not only in the length of the acyl side chain but also in the oxidation state at carbon 3 and the occasional presence of unsaturated carbon-carbon bonds. This is illustrated in three AHL structures produced by various *Vibrio* sp. [139] (Figure 12).

**Figure 13:**
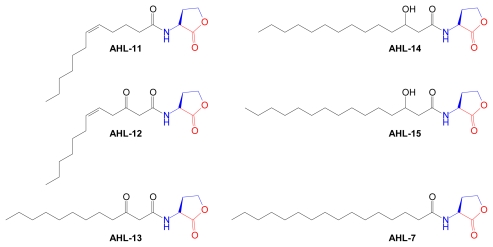
AHLs produced by marine bacteria genus *Mesorhizobium*. Structural components derived from AdoMet are color coded.

Marine gram-negative α-proteobacteria produce many novel AHL structures, such as those isolated from *Mesorhizobium* sp. [140] (Figure 13).

**Figure 14:**
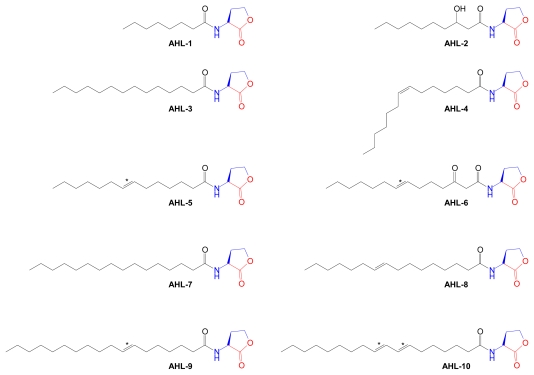
AHLs produced by marine bacteria genus *Roseobacter*. Asterisks indicate double bonds whose location and *cis-*, *trans*-orientations are unknown. Structural components derived from AdoMet are color coded.

The *Roseobacter* clade, grouped by a shared lineage, is a ubiquitous class of α-proteobacteria, [141,142]. Although *Roseobacters* can exist as free-living organisms, they frequently reside in marine habitats that promote their symbiotic associations with microalgae, corals, diatoms, oysters, etc. A significant number of *Roseobacter* strains isolated from North Sea marine habitats were found to produce complex mixtures of unusual, long-chain AHLs (Figure 14) [143].

Quorum sensing in *Roseobacters* is associated with adaptive responses, such as biofilm formation, which promote colonization of other organisms, and antibiotic production, which is presumably used for self protection [144]. Tryptantrin and thiotropocin are two known *Roseobacter*-derived antibiotics, whose production is regulated by AHL-dependent signaling [142]. Comparison of the long-chain AHLs produced by *Roseobacters* (Figure 14) and *Mesorhizobium* sp. (Figure 13) shows remarkable similarities in the acyl side chain structures. Except for **AHL-7**, which is produced by both organisms, their respective AHL signal molecules are in fact structurally distinct and allow for self discrimination.

**Figure 15:**
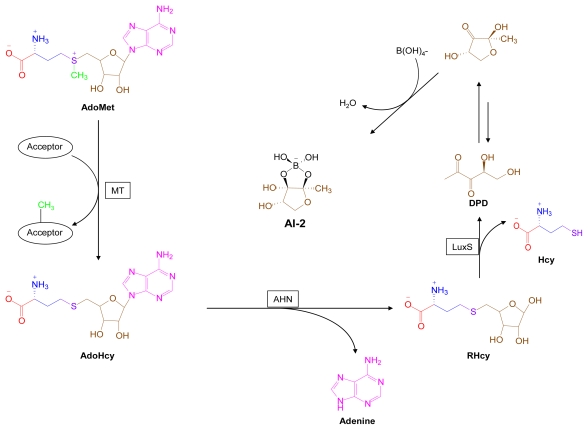
AI-2 biosynthesis. The byproduct of biological methylation pathways, AdoHcy, is enzymatically metabolized through two steps to yield DPD (*4S*-4,5-dihydroxypentane-2,3-dione), which is generated from the ribose ring of AdoMet. DPD spontaneously cyclizes and binds boric acid to form autoinducer 2 (AI-2). Structural components derived from AdoMet are color coded. MT, methyltransferase; AHN, adenosylhomocysteine nucleosidase; RHcy, ribosylhomocysteine; LuxS, *S*-ribosylhomocysteine lyase.

Autoinducer-2 (**AI-2**) is another AdoMet-derived bacterial QS signal molecule that was discovered in a marine bacterium [9,145]. The proximate precursor of **AI-2** is *S*-adenosylhomocysteine (AdoHcy), the AdoMet metabolite that is generated as the byproduct of all AdoMet-utilizing methylation reactions (Figure 15). Since AdoHcy is a potent product inhibitor of methyltransferases, conditions that allow its accumulation in cells have toxic consequences. Two pathways of AdoHcy degradation are known. One is initiated by the enzyme AdoHcy hydrolase which produces the proximate metabolites, adenosine and l-homocysteine. A second pathway of AdoHcy degradation is initiated by the enzyme AdoHcy nucleosidase, which produces the proximate metabolites, adenine and *S*-ribosylhomocysteine.

Gram-positive bacteria also utilize QS as a means to communicate and coordinate responses to a variety of environmental stimuli. However, they do not generate AHLs; instead they utilize small autoinducing peptides [146]. Although AHL production is considered to be exclusive to gram negative bacteria, both gram-positive and gram-negative bacteria are able to synthesize **AI-2**. Thus QS signaling via **AI-2** provides an avenue for interspecies communication [147].

### 3.7. N-Acylhomoserine Lactones as Templates for Anti-Infective Tetramic and Tetronic Acids

**Figure 16:**
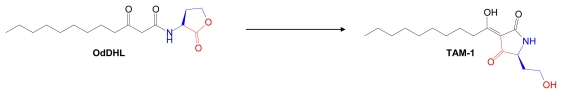
The *P. aeruginosa* QS signal, *N*-3-oxo-dodecanoyl-l-homoserine lactone, OdDHL, undergoes spontaneous degradation to form a tetramic acid [149].

Bacteria that rely on the production of QS signal molecules to coordinate expression of genetic programs, must also have signal molecule degradation pathways to effectively shut down these processes. Two major enzymatic mechanisms for AHL removal are hydrolytic opening of the lactone ring by lactonases or hydrolytic cleavage of the amide bond by acylases [148]. AHLs can also undergo a nonenzymatic chemical rearrangement to form tetramic acids (TAMs). The biological relevance of this type of AHL rearrangement was observed in *Pseudomonas aeruginosa* cultures by Kaufmann and colleagues, who isolated the expected AHL, *N*-3-oxo-dodecanoyl-l-homoserine lactone (OdDHL) as well as the corresponding tetramic acid (**TAM-1**) from the culture medium [149] (Figure 16). Notably, **TAM-1** displayed a spectrum of antibacterial activity different from that of OdDHL.

**Figure 17:**
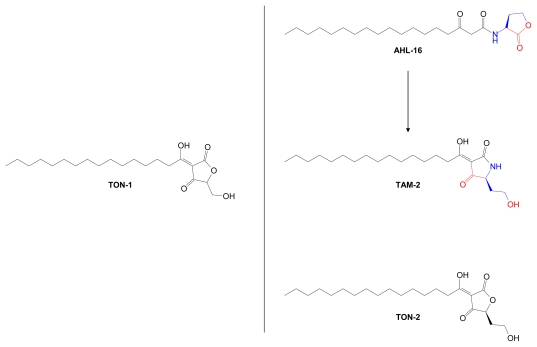
TON-1 (RK-682, 3-alkanoyl-5-hydroxymethyl tetronic acid) can serve as a retro-template for related AHL and tetramic acid structures. AHL-16, TAM-2 and TON-2 are putative structures. Structural components derived from AdoMet are color coded.

Based on these novel findings, marine-derived AHLs can be regarded as templates for the design of tetramic acid derivatives with antibacterial effects distinct from those of their parent AHLs. Similarly, the tetramic acids can serve as templates for synthesis of the related tetronic acids (TONs). In fact, 3-alkanoyl-5-hydroxymethyl tetronic acid (RK-682, **TON-1**), isolated from actinomycete strain DSM 7357, at first sight appears to be an AHL-derived tetronic acid analog [150] (Figure 17). The initial finding that **TON-1** is a potent inhibitor of tyrosine phosphatase that blocks G2/M cell cycle progression has spurred interest in analog synthesis and evaluation [150–152].

### 3.8. Marine-Derived Quorum Sensing Antagonists

**Figure 18:**
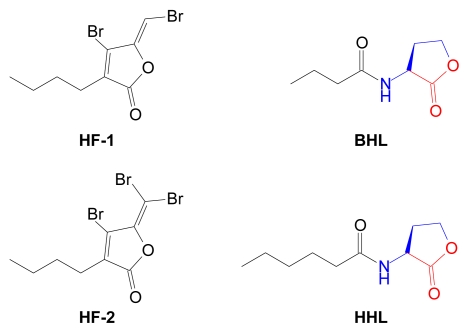
Quorum sensing antagonists. Halogenated furanones of *D. pulchra* interfere with AHL-mediated QS pathways of *Serratia liquefaciens*. Structural components derived from AdoMet are color coded. BHL, *N*-butanoyl-l-homoserine lactone; HHL, *N*-hexanoyl-l-homoserine lactone.

Marine organisms have not sat by idly while various bacteria coordinate their destruction with AHLs. Many have developed additional pathways to disrupt AHL function and QS signaling. Some marine eukaryotes produce AHL mimics that interfere with AHL-receptor binding, effectively blocking the QS signal. Halogenated furanones produced by the Australian red algae, *Delisea pulchra* are the best studied examples of naturally occurring AHL mimics [153,154] (Figure 18).

Several studies have determined that two *D. pulchra* halogenated furanones (**HF-1** and **HF-2**) disrupt the AHL-controlled swarming phenotype of *S. liquefaciens* through competitive binding of AHL receptors [155], [156]. Furthermore, binding of halogenated furanones to AHL receptors decreases receptor half life [156]. Like tetronic acids, halogenated furanones may serve as retro-templates for related AHL or tetramic acid structures with potential anti-QS and/or anti-infective activities.

### 3.9. Unusual Marine Metabolites of AdoMet

**Figure 19:**
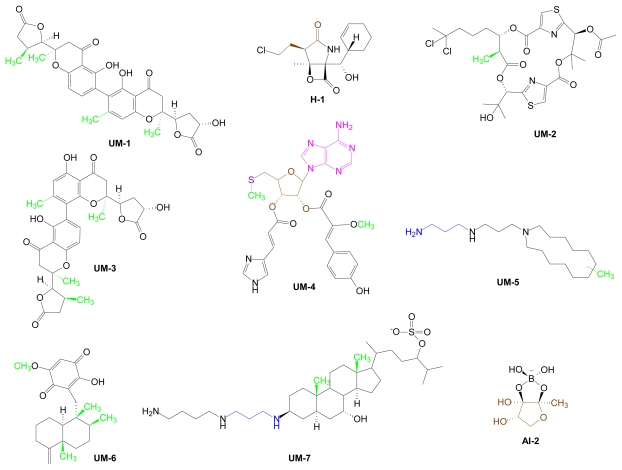
Unusual marine-derived metabolites of AdoMet (UM). Structural components derived from AdoMet are color coded.

Many AdoMet-derived compounds that were isolated from marine sources have unconventional structures and/or exhibit unusual biological properties (Figure 19). Some of these metabolites contain a polyamine backbone within a more complex chemical structure (**UM-5, UM-7**); most integrate methyl substituents within unusual structural scaffolds (**UM1** - **UM7**). From a shared chemical and biological perspective, the most extraordinary AdoMet-derived marine metabolite is, arguably, the low molecular weight boronate diester, **AI-2**, which acts as an interspecies bacterial signal molecule. Inclusion of salinosporamide A (**H-1**) in this small group of uncommon molecules reflects its novelty as the first marine metabolite known to be halogenated via an AdoMet-dependent halogenase (as noted in Section 3.4 [1]).

Motuporamines (MPAs) were first isolated from the tropical marine sponge *Xestospongia exigua* (Kirkpatrick) [157]. Their heterocyclic structures are characterized by the presence of an incorporated polyamine (*i.e.*, spermidine) tail. Additional motuporamines whose structures differ by ring size and by the presence and positions of unsaturated bonds and/or methyl substituents, have since been isolated [158]. The MPAs, **UM-5** (composite structure**)** each contain a methyl group of unassigned position. AdoMet independently serves as polyamine precursor and methyl donor for the structural components of **UM-5**. MPAs have been shown to inhibit angiogenesis and tumor cell invasiveness [158,159]. To and colleagues considered that MPAs might have similar effects on neuronal growth and motility. They studied the effects of MPA-C on neurite development in chicks and determined that this compound profoundly represses formation of the highly motile, neuronal growth cone that plays a key role in axonal outgrowth [160]. MPA-C is used as a novel neurobiological probe to study molecular mechanisms associated with neuronal outgrowth [158–161].

The sesquiterpene quinone, ilimaquinone (**UM-6**) was first isolated from the marine sponge *Hippospongia metachromia* in 1979 [162]. One of its unusual biological properties is its ability to completely vesiculate Golgi membranes and consequently affect protein transport [163]. As such, ilimaquinone has been widely used to elucidate the physiological functions of the Golgi apparatus [164,165]. Ilimaquinone has also been found to potently inhibit *S*-adenosylhomocysteine hydrolase (AHH), a key enzyme in methylation pathways [166]. AHH inhibition blocks degradation of *S*-adenosylhomocysteine, the potent product inhibitor of all AdoMet dependent methyltransferases, suggesting that AdoHcy levels are elevated in ilimaquinone-producing marine organisms. Since AdoHcy also serves as proximate precursor of the QS signal molecule, **AI-2**, “ilimaquinone-producers” in marine habitats may have an enhanced capability to synthesize **AI-2** in response to environmental stresses.

The aminosterol, squalamine (**UM-7**) is a spermidine-dihydroxycholestane-sulfate conjugate that was initially isolated from stomach extracts of the dogfish shark *Squalus acanthias* [167]. Additional aminosterols, with structural variations primarily in the sterol side chain, were subsequently isolated from the same source [168]. One of these compounds is a spermine conjugate. Squalamine is a water-soluble, broad spectrum antimicrobial, having shown activity against strains of *Escherichia coli*, *Staphylococcus aureus*, C*andida albicans* and *P. aeruginosa* [167,168]. Squalamine, which has also shown anticancer and antiangiogenic activities, has undergone phase II clinical trials against ovarian, prostate and non-small cell lung cancers [167–169].

Monodictychromones A and B (**UM-1** and **UM-3**) were found in the marine algicolous fungus, *Monodictys putredinis* by Konig and colleagues who had previously isolated a group of monomeric xanthones from the same organism [140,170]. **UM-1** and **UM-3** contain two unusual, non identical xanthone subunits and three chiral methyl substituents. **UM-1** and **UM-3** differ only by the site of their linkage [170]. The two compounds were evaluated for their ability to inhibit the activities of aromatase and cytochrome P450 1A enzymes as well as induction of NAD(P)H:quinone reductase. Both dimeric structures showed similar, but modest inhibitory effects (μM range) in these assays [170].

Hectochlorin, **UM-2** has been isolated from the marine cyanobacterium *Lyngbya majuscula* as well as the sea hare, *Bursatella leachii* [171,172]. **UM-2**’s notable biological properties include its potent stimulatory effects on actin polymerization in PtK2 (normal kidney) cells and its potent antifungal activity against *C. albicans* [171]. Gerwick and colleagues characterized the hectochlorin biosynthetic gene cluster from *L. majuscula* [173]. During these investigations, an AdoMet-dependent *C*-methyl-transferase signature motif, previously identified in the biosynthetic gene clusters of curacin A and jamaicamide from other *L. majuscula* strains, was found to be present in the hectochlorin biosynthetic gene cluster [29,173–175].

The unusual, asymmetric diester of MTA, **UM-4** was isolated from the marine ascadian, *Atriolum robustum* [175]. The 2’-ribose ester substituent of **UM-4** is derived from 3-(4-hydroxyphenyl-2-methoxyacrylic acid (HMA); the 3’-ribose ester substituent, from urocanic acid (UCA). **UM-4** was consistently inferior to MTA in receptor-specific binding assays for the A1, A2A, A2B, and A3 adenosine receptors with binding constants in the micromolar range for the A1, A2A, and A3 receptors [175]. *In silico* docking into homology models of the A1 and A3 receptors demonstrated that **UM-4** readily docks into the adenosine binding sites of both receptors, likely due to the inherent flexibility of its long chain ester substituents [175].

MTA is the enzymatic by product of three major AdoMet-dependent pathways: polyamine, ethylene and *N*-acylhomoserine lactone biosyntheses. Thus, the quantities of MTA produced by marine organisms are not insignificant. MTA, like *S*-adenosylhomocysteine, is a proximate AdoMet metabolite that is usually recycled to methionine [176,177]. Two major routes of MTA metabolism are known [178–181]. One is initiated by the enzyme MTA phosphorylase to yield adenine and 5-methyl-thioribose-1-phosphate; a second enzymatic pathway involves initial hydrolytic cleavage of MTA by one of several closely related nucleosidases, to produce adenine and 5-methylthioribose. Konig and colleagues suggested **UM-4** might be an MTA prodrug that is slowly hydrolyzed by marine esterases [175]. Expanding on this idea, **UM-4** may also be a depot form of UCA and/or HMA. UCA’s biological properties support this possibility [182]. The *trans*-isomer of UCA is biosynthesized from histidine in the outer epidermal layers of marine organisms. Subsequent exposure to UV radiation converts *trans*-UCA to the bioactive, immunosuppressive *cis*-isomer. The mechanisms associated with these biological properties, although widely studied, are not well understood [182]. Further insights into **UM-4** function may be forthcoming in the future. From a structural perspective alone, **UM-4** is, perhaps, the most bizarre AdoMet metabolite to be extracted from the oceans’ depths.

## 4. Conclusions

Marine environments continue to serve as a source of newly identified, structurally novel metabolites of AdoMet. The discovery of quorum sensing activities in gram-negative marine bacteria and the ensuing studies of QS phenomena in marine environments have been instrumental to our current understanding of the complexities of this previously unrecognized bacterial signaling network. These studies provided the first evidence of two new types of AdoMet-derived metabolites, AHLs and **AI-2**. We regard QS networks in global marine habitats as an unusually fertile source of potential, anti-infective AdoMet-derived molecules. The different types of QS-related molecules that can be used as templates for drug design include AHLs, their corresponding tetramic and tetronic acids, and halogenated furanones. Equally important drug templates will emerge from the vast array of defensive chemicals produced by marine organisms to combat the coordinated assaults of pathogenic, quorum sensing bacteria.

## Figures and Tables

**Table 1:** Examples of marine-derived, precursor-validated AdoMet metabolites.

Compound	AdoMet Metabolite	Marine Source	Reference
**PV-1**	Barbamide	*Lyngbya majuscula* (cyanobacterium)	[26–28]
**PV-2**	Dechlorobarbaramide	*Lyngbya majuscula* (cyanobacterium)	[26]
**PV-3**	Curacin A	*Lyngbya majuscula* (cyanobacterium)	[29]
**PV-4**	Brassicasterol	*Bugula neritina* (bryozoan)	[30]
**PV-5**	Gorgosterol	*Isis hippuris* (coral)	[31]
**PV-6**	Mutasterol	*Xestspongia muta* (sponge)	[32]
**PV-7**	Prugosene A1	*Penicillium rugulosum* (sponge-derived fungus)	[33]
**PV-8**	Phomopsidin	*Phomopsis sp* (fungus)	[34]
**PV-9**	Sorbicillactone A	*Penicillium chrysogenum* (sponge)	[35]
**PV-10**	Sorbicillactone B	*Penicillium chrysogenum* (sponge)	[35]

**Table 2:** Linear aliphatic polyamines derived from *Thermus thermophilus* [46,52]. AdoMet-derived terminal aminopropyl groups are shown in blue.

Compound	Polyamine	Structure
**PA-1**	Diaminopropane	H_2_N(CH_2_)**_3_**NH_2_
**PA-2**	Putrescine	H_2_N(CH_2_)**_4_**NH_2_
**PA-3**	Cadaverine	H_2_N(CH_2_)**_5_**NH_2_
**PA-4**	Norspermidine	H_2_N(CH_2_)**_3_**NH(CH_2_)**_3_**NH_2_
**PA-5**	Spermidine	H_2_N(CH_2_)**_3_**NH(CH_2_)**_4_**NH_2_
**PA-6**	Homospermidine	H_2_N(CH_2_)**_4_**NH(CH_2_)**_4_**NH_2_
**PA-7**	Thermine	H_2_N(CH_2_)**_3_**NH(CH_2_)**_3_**NH (CH_2_)**_3_**NH_2_
**PA-8**	Spermine	H_2_N(CH_2_)**_3_**NH(CH_2_)**_4_**NH (CH_2_)**_3_**NH_2_
**PA-9**	Thermospermine	H_2_N(CH_2_)**_3_**NH(CH_2_)**_3_**NH(CH_2_)**_4_** NH_2_
**PA-10**	Homospermine	H_2_N(CH_2_)**_3_**NH(CH_2_)**_4_**NH(CH_2_)**_4_** NH_2_
**PA-11**	Caldopentamine	H_2_N(CH_2_)**_3_**NH(CH_2_)**_3_**NH(CH_2_)**_3_**NH (CH_2_)**_3_**NH_2_
**PA-12**	Thermopentamine	H_2_N(CH_2_)**_3_**NH(CH_2_)**_3_**NH(CH_2_)**_4_**NH (CH_2_)**_3_**NH_2_
**PA-13**	Homocaldopentamine	H_2_N(CH_2_)**_3_**NH(CH)**_3_**NH(CH_2_)**_3_**NH(CH_2_)**_3_**NH(CH_2_)_4_NH_2_
**PA-14**	Caldohexamine	H_2_N(CH_2_)**_3_**NH(CH_2_)**_3_**NH(CH_2_)**_3_**NH(CH_2_)**_3_**NH (CH_2_)_3_NH_2_
**PA-15**	Homocaldohexamine	H_2_N(CH_2_)**_3_**NH(CH_2_)**_3_**NH(CH_2_)**_3_**NH(CH_2_)**_3_**NH(CH_2_)_4_NH_2_

**Table 3:** Examples of marine-derived polyamine conjugates (PACs).

Compound	Polyamine Conjugate	Marine Source	Reference
**PAC-1**	Acarnidines	*Acarnus erithacus* (sponge)	[58]
**PAC-2**	*N*-trimethylSpd FAE*	*Sinularia brongersmai* (coral)	[59]
**PAC-3**	Sinulamide	*Sinularia***s**p. 1 (coral)	[59,60]
**PAC-4**	Penaramide A	*Penares* aff. *Incrustans* (sponge)	[61]
**PAC-5**	Crambescidin 800	*Crambe crambe* (sponge)	[53,62]
**PAC-6**	Ptilomycalin A	*Hemimycale* sp (sponge)	[53,62]
**PAC-7**	Pseudoceratidine	*Pseudoceratina purpurea* (sponge)	[63]
**PAC-8**	Spermatinamine	*Pseudoceratina* sp. (sponge)	[64]
**PAC-9**	Petrobactin 1	*Bacillus anthracis* str. Sterne (bacterium)	[65]

* *N*-trimethylspermidine fatty acid ester.

**Table 4:** Examples of marine-derived methylated purines (MP).

Compound	Purine	Marine Source	Ref.
**MP-1**	2-Hydroxy-1’-methylzeatin	Green algae and blue coral	[89]
**MP-2**	Nigricine 4	*Petrosia nigricans* (sponge)	[90]
**MP-3**	1,3-Dimethylguanine	*Botrylloides leachi* (acidian)	[91]
**MP-4**	3,7-Dimethylguanine	*Zyzzya fuliginosa* (sponge)	[92]
**MP-5**	1,3,7-Trimethylguanine	*Latrunculia brevis* (sponge) *Eudistoma maculosum* (ascidian)	[93] [94]
**MP-6**	*N*^2^,*N*^2^,*N*^7^-Trimethylguanine	*Lissoclinum notti* (ascidian)	[95]
**MP-7**	6-Methoxy-7-methyl-8-oxoguanine	*Symplegma rubra* (ascidian)	[96]
**MP-8**	1,3-Dimethylisoguanine	*Amphimedon viridis* (sponge)	[97]
**MP-9**	3,7-Dimethylisoguanine	*Agelas longissima* (sponge)	[98]
**MP-10**	1,3,7-Trimethylisoguanine	*Pseudodistoma cereum* (ascidian)	[99]
**MP-11**	1,3-Dimethyl-8-oxoisoguanine	*Pseudodistoma cereum* (ascidian)	[100]
**MP-12**	3-Methyl-6-methylamino-2-methylimino-9H-purine	*Sagartia troglodytes* Price (sea anemone)	[101]
**MP-13**	2-Hydroxy-6-methylaminopurine	Green algae and blue coral	[89]
**MP-14**	1-Methyl-6-iminopurine	*Hymeniacidon* Grant (sponge)	[102]
**MP-15**	1,9-Dimethyl-6-imino-8-oxopurine	*Hymeniacidon sanguinea* Grant (sponge)	[102]
**MP-16**	Caissarone	*Bunodosoma-Caissasum* (sea-anemone)	[103]
**MP-17**	1-Methylherbipoline	*Jaspis* sp (sponge)	[104]
**MP-18**	Mucronatine	*Stryphnus mucronatus* (sponge)	[105]
